# 5'-Chloro-5'-deoxy-(±)-ENBA, a Potent and Selective Adenosine A_1_ Receptor Agonist, Alleviates Neuropathic Pain in Mice Through Functional Glial and Microglial Changes without Affecting Motor or Cardiovascular Functions

**DOI:** 10.3390/molecules171213712

**Published:** 2012-11-22

**Authors:** Livio Luongo, Riccardo Petrelli, Luisa Gatta, Catia Giordano, Francesca Guida, Patrizia Vita, Palmarisa Franchetti, Mario Grifantini, Vito de Novellis, Loredana Cappellacci, Sabatino Maione

**Affiliations:** 1 Section of Pharmacology “L. Donatelli”, Department of Experimental Medicine, Faculty of Medicine and Surgery, II University of Naples, via Costantinopoli 16, 80138 Naples, Italy; Email: livio.luongo@gmail.com (L.L.); luisa.gatta@hotmail.it (L.G.); catiagiordano@hotmail.com (C.G.); francesca-guida@libero.it (F.G.); vito.denovellis@unina2.it (V.N.); 2 School of Pharmacy, Medicinal Chemistry Unit, University of Camerino, Via S. Agostino 1, 62032 Camerino, Italy; Email: riccardo.petrelli@unicam.it (R.P.); patrizia.vita@unicam.it (P.V.); palmarisa.franchetti@unicam.it (P.F.); mario.grifantini@unicam.it (M.G.)

**Keywords:** A_1_ adenosine receptor, neuropathic pain, allodynia, glia

## Abstract

This study was undertaken in order to investigate the effect of chronic treatment with 5′-chloro-5′-deoxy-(±)-ENBA, a potent and highly selective agonist of human adenosine A_1_ receptor, on thermal hyperalgesia and mechanical allodynia in a mouse model of neuropathic pain, the Spared Nerve Injury (SNI) of the sciatic nerve. Chronic systemic administration of 5′-chloro-5′-deoxy-(±)-ENBA (0.5 mg/kg, i.p.) reduced both mechanical allodynia and thermal hyperalgesia 3 and 7 days post-SNI, in a way prevented by DPCPX (3 mg/kg, i.p.), a selective A_1_ adenosine receptor antagonist, without exerting any significant change on the motor coordination or arterial blood pressure. In addition, a single intraperitoneal injection of 5′-chloro-5′-deoxy-(±)-ENBA (0.5 mg/kg, i.p.) 7 days post-SNI also reduced both symptoms for at least two hours. SNI was associated with spinal changes in microglial activation ipsilaterally to the nerve injury. Activated, hypertrophic microglia were significantly reduced by 5′-chloro-5′-deoxy-(±)-ENBA chronic treatment. Our results demonstrated an involvement of adenosine A_1_ receptor in the amplified nociceptive thresholds and in spinal glial and microglial changes occurred in neuropathic pain, without affecting motor coordination or blood pressure. Our data suggest a possible use of adenosine A_1_ receptor agonist in neuropathic pain symptoms.

## 1. Introduction

Neuropathic pain is a devastating disease that can seriously affect the quality of life. It represents a debilitating consequence of peripheral or central nervous system (CNS) injury which shows an amplified transmission of nociceptive messages [1,2]. As a consequence, noxious stimuli are perceived as more painful (hyperalgesia), and normal, harmless stimuli elicit pain (allodynia). Therefore, neuropathic pain represents a real nervous system dysfunction characterized by neurophysiological changes that are still poorly understood. Recent reports have highlighted the role of microglia and astrocytes in spinal plasticity and in neuropathic pain establishment [3,4,5,6]. The knowledge of molecular changes at the spinal level could be the clue to neuropathic pain treatment. Adenosine is a regulatory nucleoside that can be generated in response to cellular stress and tissue damage as well as during episodes of tissue hypoxia or inflammation. It acts through specific G-protein coupled receptors that have been classified into four subtypes (A_1_, A_2A_, A_2B_ and A_3_) on the basis of their structures and signal transduction systems [7]. The anti-inflammatory role of adenosine has largely been investigated in macrophages, where it has been shown to inhibit the production of several pro-inflammatory cytokines such as IL-1b and to enhance the release of the anti-inflammatory cytokine IL-10 [8]. Activation of adenosine A_1_ receptor (A_1_AR) produces antinociception, whereas activation of A_2A_/A_2B_ receptors produces pro-nociceptive or pain-enhancing effects at the peripheral sensory nerve terminal level in rodents [9,10]. Hence, antinociception may be induced by selective adenosine A_1_AR agonists, or by selective adenosine A_2A_/A_2B_ receptor antagonists. The considerable therapeutic potential of A_1_AR agonists has prompted decades of work which has led to the discovery of many selective compounds [11,12,13,14,15]. There is evidence that A_1_AR agonists produce antinociception at a spinal cord level [16,17], as well as at the supraspinal level [18]. In addition, Wu and coworkers demonstrated that the nociceptive response is increased in mice lacking the A_1_AR [19]. Indeed, it has recently been reported that *N*^6^-cyclopentyladenosine (CPA), a selective A_1_AR agonist, is a potent and effective analgesic in acute arthritis and neuropathic pain models [20]. Despite these encouraging results, several side effects of CPA preclude its clinical use.

In fact, the analgesic effect of A_1_AR agonists is frequently associated with depression of arterial blood pressure and bradycardia [20,21,22]. Beside the analgesic effect of CPA mediated by A_1_AR stimulation, other studies suggest a neuroprotective role [23,24]. Moreover, it is intriguing that A_1_AR is highly expressed on both macrophages and neurons in the CNS [25]. In the latter, A_1_AR is coupled to the activation of K^+^ channels [26] and inhibition of Ca^2+^ channels [27]; both mechanisms that attenuate neuronal excitability.

It is worth noting that two A_1_-selective agonists, GR79236 and GW493838, have been clinically evaluated for the treatment of neuropathic pain. GR79236 has analgesic and anti-inflammatory actions in humans and animals, whereas GW493838 has been evaluated in phase 2 clinical trials for pain management [28]. Moreover a recent paper published by Zylka *et al. *[29] reported a novel series of potent and selective A_1_AR agonists, which showed potent antinociceptive effects and lack of cardiovascular side effects.

Based on these findings, in this study we have evaluated the anti-neuropathic properties of a potent and highly selective agonist of human adenosine A_1_ receptor, the compound 5′-chloro-5′-deoxy-*N*^6^-(±)-*endo*-norborn-2-yl)adenosine [5′-chloro-5′-deoxy-(±)-ENBA, 5′Cl5'd-(±)-ENBA, *h*A_1_AR (*K*_i_) = 0.51 nM, *h*A_2A_AR (*K*_i_) = 1,340 nM, *h*A_2B_AR (*K*_i_) = 2,740 nM, *h*A_3_AR (*K*_i_) = 1,290 nM, EC_50_ = 6.75 nM], whose pharmacokinetic and pharmacodynamic profiles as well as its acute nocifensive effect have been investigated in the formalin test in mice [15]. For this purpose we used the spared nerve injury (SNI) of the sciatic nerve in mice, a model of neuropathic pain which has been extensively validated by several research groups [30]. Moreover, we also have evaluated the expression and a possible role of the A_1_AR on glial cells. Behavioural, cardiovascular and immunohistochemical approaches have been used to investigate the effect of prolonged treatment with 5′Cl5′d-(±)-ENBA or saline on: (i) mechanical allodynia and thermal hyperalgesia; (ii) motor coordination, blood pressure and heart rate; (iii) glial and microglial activation in the spinal cord. 

## 2. Results

### 2.1. 5′Cl5′d-(±)-ENBA Reduced Thermal Hyperalgesia and Mechanical Allodynia in SNI Mice and Did Not Affect Motor or Cardiovascular Functions

**Figure 1:**
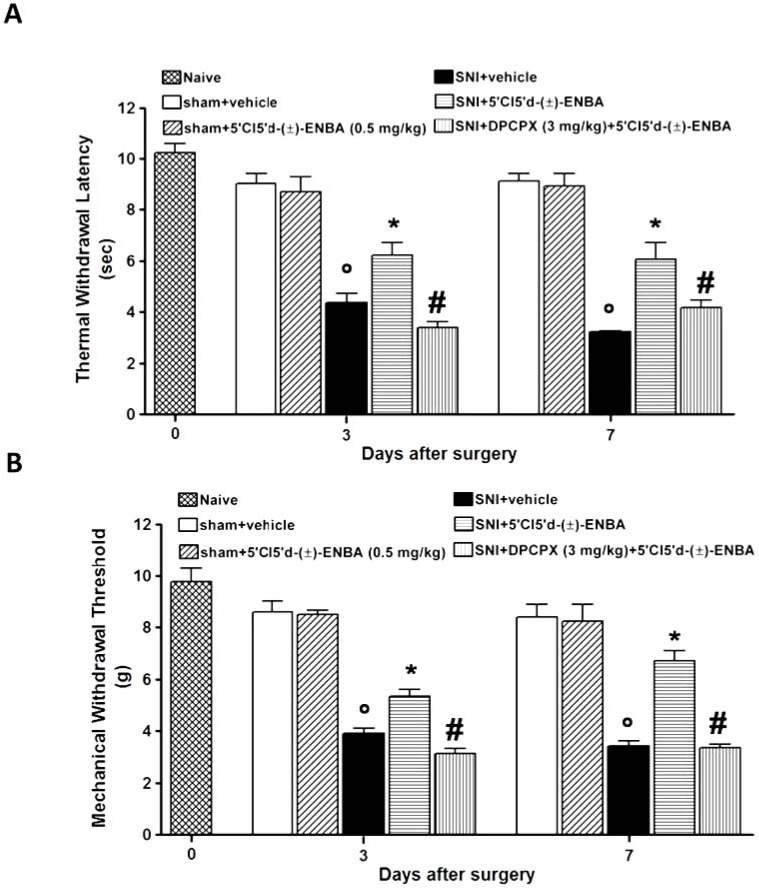
Chronic treatment with 5′Cl5′d-(±)-ENBA reduces mechanical allodynia (**A**) and thermal hyperalgesia (**B**). SNI mice showed mechanical allodynia and thermal hyperalgesia only at the ipsilateral sides of the nerve injury at 3 and 7 days post-surgery. Administrations of 5′Cl5'd-(±)-ENBA (0.5 mg/kg), once per day, reduced reflex withdrawal responses to mechanical and noxious thermal stimuli in SNI mice and this effect was prevented by pre-treatment with DPCPX (3 mg/kg) (**A**, **B**). Data represent the mean of the mechanical paw withdrawal threshold and the thermal paw withdrawal latency for mechanical allodynia and thermal hyperalgesia respectively, of six randomly selected mice for each histogram. ° *p* < 0.05 *vs.* sham operated mice or naive mice, * *p* < 0.05 *vs*. SNI mice, # *p *< 0.05 *vs*. 5′Cl5'd-(±)-ENBA treated SNI mice.

Spared nerve injury (SNI) was associated with the development of ipsilateral mechanical and thermal hypersensitivity which were monitored up to 7 days after surgery (Figure 1A,B). Both contralateral and sham operated thresholds remained unaltered. Chronic administration of 5′Cl5′d-(±)-ENBA (0.5 mg kg^−^^1^, i.p.) from day 1 to day 7 reduced mechanical allodynia at 3 and 7 days after nerve injury (Figure 1A). Furthermore, thermal hyperalgesia was reduced by 5′Cl5′d-(±)-ENBA 3 and 7 days after SNI induction (Figure 1B). The analgesic effect of 5′Cl5′d-(±)-ENBA was no greater at a dose of 0.1 mg kg^−^^1^ (data not shown) while at 0.5 mg kg^−^^1^ it was statistically significant. The anti-allodynic and anti-hyperalgesic effects of 5′Cl5′d-(±)-ENBA were prevented by the concurrent treatment with the A_1_AR antagonist, DPCPX (3 mg kg^−^^1^, i.p.) (Figure 1A,B) which *per se* did not affect mechanical and thermal latency (data not shown).

**Figure 2:**
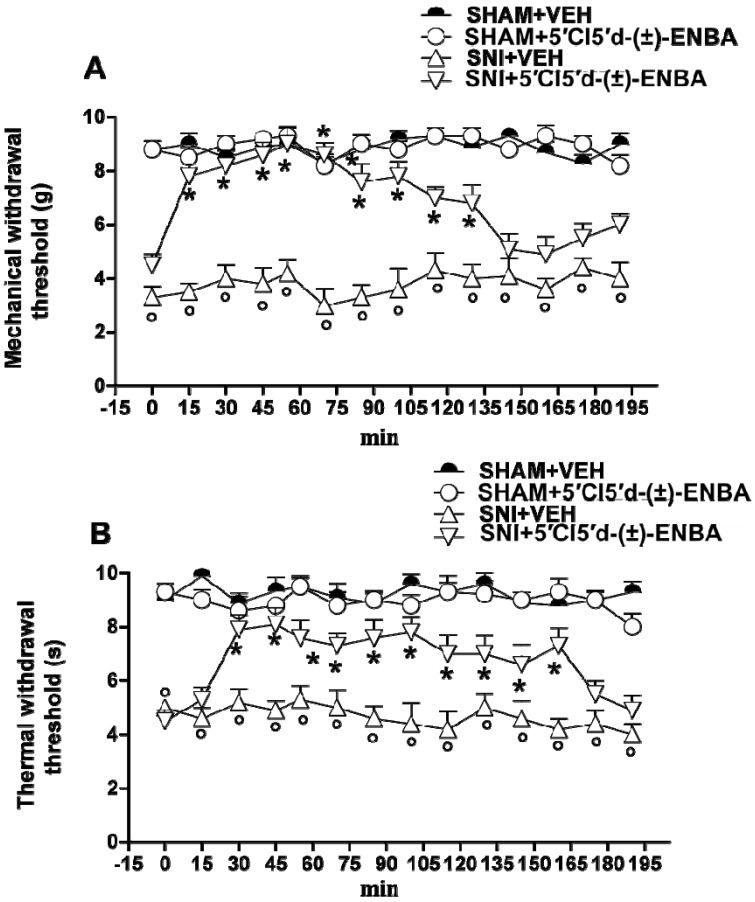
A single administration (7 days after SNI) of 5′Cl5'd-(±)-ENBA reduces mechanical allodynia (**A**) and thermal hyperalgesia (**B**). 5′Cl5'd-(±)-ENBA (0.5 mg/kg) was effective in reducing the appearance of mechanical allodynia and thermal hyperalgesia in SNI mice. ° *p* < 0.05 *vs*. sham operated mice or naive mice, * p < 0.05 *vs*. SNI mice.

DMPX, a selective A_2A_AR antagonist, was unable to block the antiallodynic effect of 5′Cl5′d-(±)-ENBA (data not shown). Besides chronic treatment, a single administration of 5′Cl5′d-(±)-ENBA (0.5 mg kg^−^^1^) also reduced mechanical allodynia and thermal hyperalgesia for at least 2 h of monitoring (Figure 2A,B).

**Figure 3:**
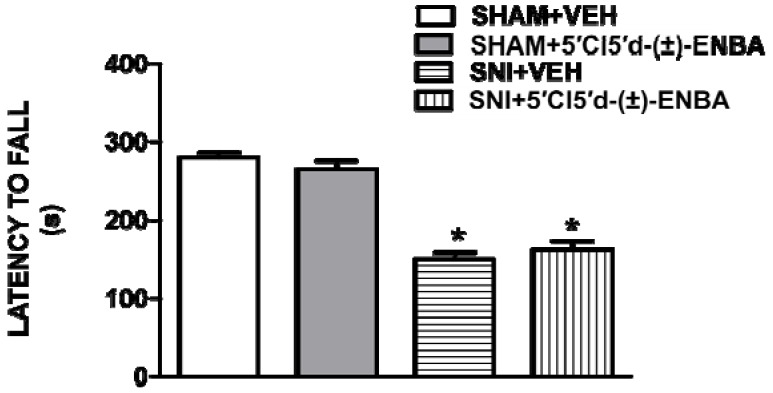
A single administration of 5′Cl5'd-(±)-ENBA does not affect the motor performance in Rotarod test. SNI mice showed an impaired motor activity 7 days post injury. Single administrations of 5′Cl5'd-(±)-ENBA (0.5 mg/kg) did not influence the locomotive activity as compared with sham or SNI mice. * p < 0.05 *vs*. sham mice. Data are expressed as mean of repeated measures performed every 30 min for 5 h.

Moreover, we performed the Rotarod test to evaluate whether the antinociceptive dose of the 5′Cl5′d-(±)-ENBA had any effect on motor coordination. 5′Cl5′d-(±)-ENBA treatment did not modify motor coordination as compared to vehicle-treated SNI mice (Figure 3). However, SNI mice showed an impairment in the motor activity due to the rescission of the motor component of the sciatic nerve. Furthermore, 5′Cl5′d-(±)-ENBA treatment at the effective dose did not modify blood pressure or heart rate. Finally, 5′Cl5′d-(±)-ENBA (0.5 mg/Kg, i.p.) did not significantly change arterial blood pressure or heart rate in unanaesthetized sham and SNI mice (Table 1).

**Table 1:** Effects of an antiallodynic dose of 5′Cl5′d-(±)-ENBA on systolic blood pressure (mmHg) and heart rate (BPM) in Sham and SNI mice.

	Sham + Vehicle	Sham + 5′Cl5'd-(±)-ENBA	SNI + Vehicle	SNI + 5′Cl5'd-(±)-ENBA
Systolic Blood Pressure (mmHg)	113 ± 2.4	111.3 ± 1.9	110.4 ± 2.1	108.4 ± 2.6
Heart Rate (BPM)	520.12 ± 3.9	518.12 ± 2.9	512.31 ± 3.4	515.15 ± 2.3

**Systolic Blood Pressure (mmHg): Sham + 5′Cl5'd-(±)-ENBA *vs.* Sham +Vehicle** F_(1–10)_ = 0.31, *p* = 0.591, *p* > 0.05; **SNI + Vehicle *vs.* Sham +Vehicle** F_(1-10)_ = 0.66, *p* = 0.434, *p* > 0.05; **SNI + 5′Cl5'd-(±)-ENBA *vs.* SNI + Vehicle** F_(1–10)_ = 0.36, P = 0.563, *p* > 0.05; **Heart Rate (BPM): Sham + 5′Cl5'd-(±)-ENBA *vs.* Sham + Vehicle** F_(1–10)_ = 0.17, *p* = 0.689, *p* > 0.05; **SNI + Vehicle *vs.* Sham + Vehicle** F_(1–10)_ = 2.28, *p* = 0.162, *p* > 0.05; **SNI + 5′Cl5'd-(±)-ENBA *vs*. SNI + Vehicle** F_(1–10)_ = 0.48, *p* = 0.505, *p* > 0.05.

### 2.2. 5′Cl5′d-(±)-ENBA-Induced Analgesia is Associated with a Reduction in Glial and Microglial Activation 7 Days after SNI Induction

**Figure 4:**
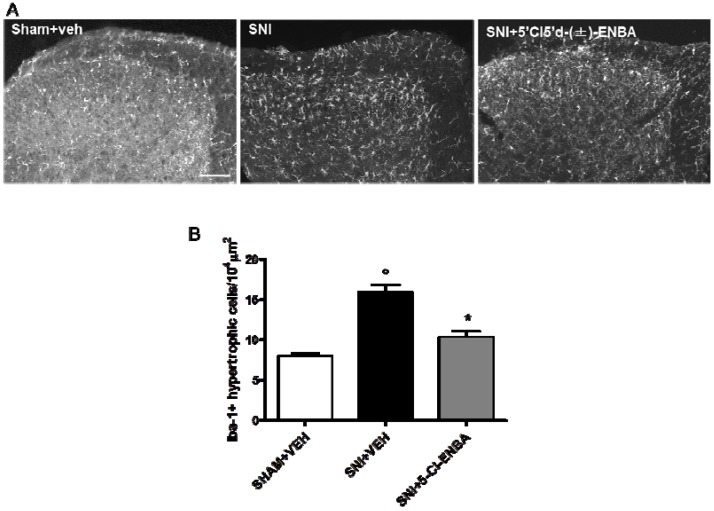
(**A**) 5′Cl5'd-(±)-ENBA reduces microglial activation in the dorsal horn of SNI mice. SNI induction increased the number of activated microglia, estimated by ratio processes length/soma diameter. (**B**) Iba-1 immunoreactivity (Iba-1-ir) in the ipsilateral dorsal horn 7 days post-SNI and after 5′Cl5′d-(±)-ENBA treatment. Quantitative analysis of spinal cord sections shows significantly increased numbers of activated microglia cells in the ipsilateral dorsal horn of SNI mice as compared with sham-operated mice. Chronic 5′Cl5′d-(±)-ENBA treatment reduced the number of activated microglia as compared with SNI mice. Data represent mean ± S.E.M., n = 3 mice per group. ° *p* < 0.05 *vs*. sham mice, * *p* < 0.05 *vs*. SNI mice. ANOVA, *post hoc* Tukey. Scale bars = 100 μm.

Microglia cell number increased in the ipsilateral dorsal horn of the spinal cord (L4-L6) in SNI mice as compared to sham operated mice (Figure 4A). Morphological analysis revealed that 5′Cl5′d-(±)-ENBA treatment reduced the number of activated microglia cells in the ipsilateral dorsal horn of SNI mice (Figure 4B). 

**Figure 5:**
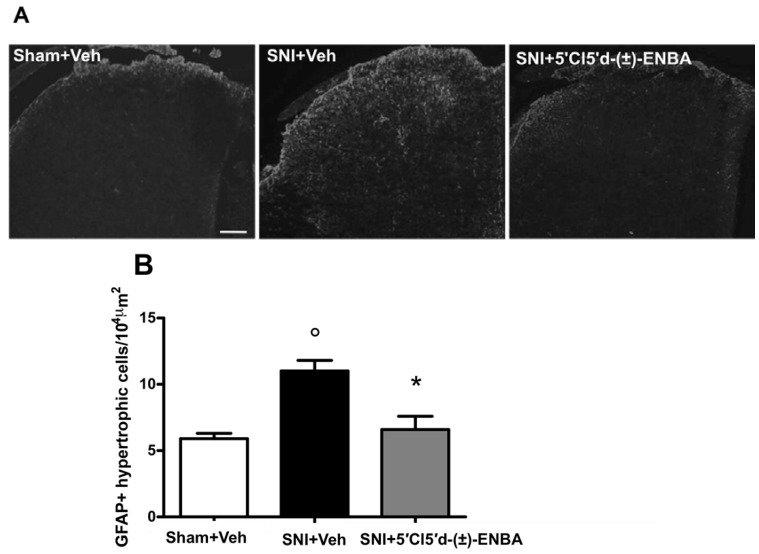
(**A**) 5′Cl5′d-(±)-ENBA reduces the number of hypertrophic astrocytes in the dorsal horn of SNI mice. GFAP (GFAP-ir) is shown in the ipsilateral dorsal horn 7 days post-SNI and after 5′Cl5′d-(±)-ENBA treatment. Quantitative analysis of spinal cord sections shows significantly increased numbers of hypertrophic astrocytes in the ipsilateral dorsal horn of SNI mice as compared with sham-operated mice. (**B**) Chronic 5′Cl5′d-(±)-ENBA treatment reduced the number of hypertrophic astrocytes as compared with SNI mice. Data represent mean ± S.E.M., n = 3 mice per group. ° *p* < 0.001 *vs*. sham mice, * *p* < 0.001 *vs*. SNI mice. ANOVA, *post hoc* Tukey. Scale bars = 100 μm.

Glial cell number increased in the ipsilateral dorsal horn of the spinal cord (L4-L6) in SNI mice as compared to sham operated mice (Figure 5A). Morphological analysis, performed following the criteria previously described [6,31], revealed that 5′Cl5′d-(±)-ENBA treatment reduced the number of activated astrocytes in the ipsilateral dorsal horn of SNI mice (Figure 5B). 

### 2.3. A_1_AR is over-Expressed in Glial Cells after Peripheral Nerve Injury

**Figure 6:**
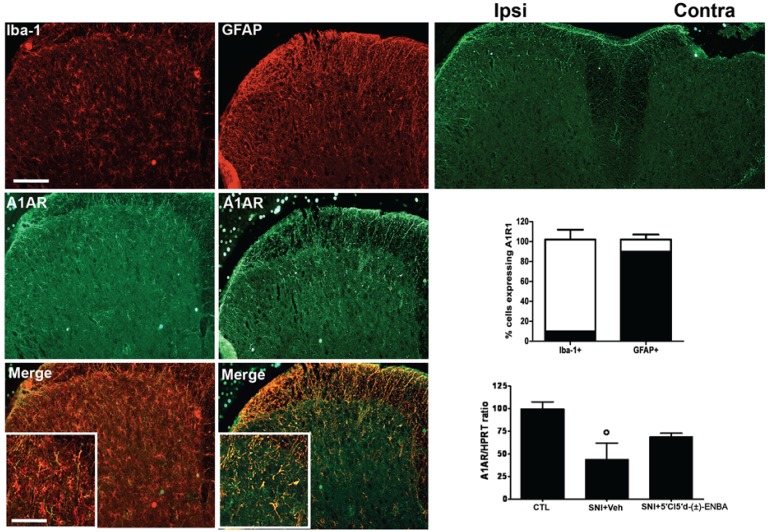
A_1_AR positive profiles are upregulated 7 days after injury in the ipsilateral dorsal horn of the spinal cord. A_1_AR positive profiles are expressed by GFAP positive astrocytes rather than Iba-1 labeled microglial cells as revealed by double staining and the related quantification. Data represent mean ± S.E.M., n = 3 mice per group. Scale bars = 100 μm.

A_1_AR staining revealed that this receptor was up-regulated in the ipsilateral dorsal horn of the spinal cord 7 days post-SNI in cells which were identified mostly as GFAP labeled astrocytes rather than microglia (Figure 6). 

## 3. Discussion

In this study, we have demonstrated the anti-allodynic and anti-hyperalgesic properties of the potent and highly selective human A_1_AR agonist 5′Cl5′d-(±)-ENBA on the SNI model of neuropathic pain in mice. Moreover, we also proved the antigliotic effect of 5′Cl5′d-(±)-ENBA on astrocytes and microglia by using an *ex vivo *immunohistochemical approach. Chronic administration of 5′Cl5′d-(±)-ENBA (0.5 mg kg^−1^) significantly reduced mechanical allodynia and thermal hyperalgesia up to 7 days after nerve injury. 

The anti-allodynic/hyperalgesic effect of the drug seemed to be mediated by the A_1_AR since the A_1_AR antagonist DPCPX reverted the 5′Cl5′d-(±)-ENBA antiallodynic effect. Moreover, a single dose of the drug 7 days after nerve injury also reduced neuropathic symptoms for at least 2 h after drug injection. Accordingly, it has been demonstrated that 5′Cl5′d-(±)-ENBA, like other selective A_1_AR agonists, reduced formalin-induced nociceptive behaviour in mice [15,18]. This acute and transient effect could be explained assuming a peripheral or spinal action of the 5′Cl5′d-(±)-ENBA on A_1_AR expressed by immune cells [32]. Indeed, we cannot exclude that an overall anti-inflammatory effect of A_1_AR agonists at the peripheral site [33], or at the spinal cord level, could transiently ameliorate the neuropathic symptoms. This hypothesis could be corroborated by the new evidence highlighting the involvement of infiltrating spinal cord lymphocytes in the establishment or maintenance of neuropathic pain [34], as well as by the ability of a specific class of T-lymphocytes to sustain the protective glial phenotype [35]. Importantly, in this study, the 5′Cl5′d-(±)-ENBA treatment did not affect either motor coordination, blood pressure or heart rate. Thus, the lack of cardiovascular side effects of our potent and selective A_1_AR agonist could indicate that it is scarcely acting on the peripheral A_1_AR which, in fact, limits the clinical use of similar drugs despite their considerable analgesic properties [20,21,22]. Indeed, at the dose we used, 5′Cl5′d-(±)-ENBA could mainly target cells highly-expressing the A_1_AR, such as the spinal cord astrocytes, and/or also microglia in the ipsilateral side of the nerve injury. It has been demonstrated that SNI determines an enhancement of activated astrocytes and microglia in the ipsilateral dorsal horn in mice [6,36], and we show here that chronic treatment with 5′Cl5′d-(±)-ENBA significantly reduces the number of activated glial cells identified as profiles presenting the diameter of the soma ≥ than the processes length as compared to the vehicle-treated SNI mice. The reduction of the activated astrocytes in the ipsilateral dorsal horn of spinal cord could be due to the direct action of 5′Cl5′d-(±)-ENBA on the Gi-coupled A_1_AR over-expressed on astrocytes, which we found in the present study together with a combined peripheral action on the T-lymphocytes [37] and/or a direct neuronal A_1_AR activation. Importantly, the drug we have used in this study is very selective for both human and murine A_1_AR. Thus, the reduction of the peripheral side effects that we found with 5′Cl5′d-(±)-ENBA in mice might be predictable also in humans since the homology of the human and murine aminoacid sequence is estimated to be about 95% (see Supplementary Figure 1).

## 4. Experimental

### 4.1. Animals

Male CD-1 mice (35–40 g) were housed as three *per* cage under controlled illumination (12–12 h light-dark cycle; light on 06:00 h) and environmental conditions (room temperature 20–22 °C, humidity 55–60%) for at least one week before the commencement of experiments. Mouse chow and tap water were available *ad libitum*. The experimental procedures were approved by the Animal Ethics Committee of the Second University of Naples. Animal care was in compliance with the IASP and European Community (E.C. L358/1 18/12/86) guidelines on the use and protection of animals in experimental research. All efforts were made to minimise animal suffering and to reduce the number of animals used.

### 4.2. Spared Nerve Injury

Mononeuropathy was induced according to the method of Schields *et al.* [30]. Mice were anaesthetised with sodium pentobarbital (50 mg/kg, i.p.). The sciatic nerve was exposed at the level of its trifurcation into sural, tibial and common peroneal nerves. The sural and common peroneal nerves were ligated tightly with 5.0 silk thread and then transected just distal to the ligation, leaving the tibial nerve intact. Sham mice were anaesthetised, the sciatic nerve was exposed at the same level, but not ligated.

### 4.3. Nociceptive Behaviour

Thermal hyperalgesia was evaluated by using the Plantar Test Apparatus (Ugo Basile, Varese, Italy). On the day of the experiment each animal was placed in a plastic cage (22 cm × 17 cm × 14 cm; length × width × height) with a glass floor. After a 30 min habituation period, the plantar surface of the hind paw was exposed to a beam of radiant heat through the glass floor. The radiant heat source consisted of an infrared bulb (Osram halogen-Bellaphot bulb; 8 V, 50 W). A photoelectric cell detected light reflected from the paw and turned off the lamp when paw movement interrupted the reflected light. The paw withdrawal latency was automatically displayed to the nearest 0.1 s; the cut-off time was 20 s in order to prevent tissue damage.

Mechanical allodynia was measured by using the Dynamic Plantar Aesthesiometer (Ugo Basile). Mice were allowed to move freely in one of the two compartments of the enclosure positioned on the metal grid surface. A mechanical stimulus was delivered to the plantar surface of the hind paw of the mouse through the metal grid by an automated steel filament exerting an increasing force of 3 grams per second. The force inducing paw withdrawal was recorded to the nearest 0.1 g. Nociceptive responses for thermal and mechanical sensitivity (thermal withdrawal latency and mechanical withdrawal threshold) were measured in seconds and grams. Baseline thresholds were determined 6 days before commencing with the treatments. Each mouse served as its own control, the responses being measured both before and after surgical procedures. The observer was blind to the treatments.

### 4.4. Motor Coordination Behaviour

Neurological functions and motor coordination were evaluated by the Rotarod motor test (Ugo Basile), which consists of putting the mouse on a rotary cylinder in order to measure the time (in second) of its equilibrium before falling. The cylinder is subdivided into five sections, allowing the screening of at least five animals per test (one per section), simultaneously. Below the cylinder there is a platform which is in turn subdivided into five plates (corresponding to the five sections) each of which is connected to a magnet that, when activated by the fall of the mouse onto the plate, allows the length of time spent on the cylinder to be recorded. After a period of adaptation of 30 s, the spin speed gradually increased from 5 to 40 rpm for the maximum time of 5 min. On the same day, the animals were analyzed by two separate tests at an interval of time of 1 h. The experiment was performed for each group of animals: the day prior to the surgical procedure, the day before the behavioural tests in order to avoid any unnecessary stress, the day before the drug administration, and from day 6 after SNI. The length of time spent by the mouse on the cylinder was expressed as latency (s).

### 4.5. Non-Invasive Blood Pressure and Heart Rate Measurements

Systolic blood pressure (SBP) was measured in restrained awake sham and SNI mice by means of the tail-cuff method (Blood Pressure Analysis System, BP-2000, Visitech System, Physiological Research Instruments, Apex, NC, USA). Mice were accustomed to the blood pressure measurement device for 5–6 days. 5′Cl5′d-(±)-ENBA was administered intraperitoneally (i.p.) and measurements were undertaken before and after drug administration. Results were expressed as means ± SEM and *p* < 0.05 was considered statistically significant. 

### 4.6. Spinal Cord Immunohistochemistry

Under pentobarbital anaesthesia, animals were transcardially perfused with saline solution followed by 4% paraformaldehyde in 0.1 M phosphate buffer. The lumbar spinal cord was excised, post fixed for 3 h in the perfusion fixative, cryoprotected for 72 h in 30% sucrose in 0.1 M phosphate buffer and frozen in O.C.T. embedding compound. Transverse sections (20 μm) were cut using a cryostat and thaw-mounted onto glass slides. Slides were incubated overnight with primary antibody solutions for the astrocytic cell marker GFAP (rabbit anti-glial fibrillary acidic protein; 1:1,000; Dako, Milan, Italy) or microglial cell marker Iba-1 (polyclonal rabbit anti ionized calcium binding adapter molecule-1, Wako, Neuss, Germany) alone or in combination with the primary antibody against A_1_AR (polyclonal goat anti A_1_AR, Santa Cruz Biotechnology Inc., Santa Cruz, CA, USA). Following incubation sections were washed and incubated for 3 h with secondary antibody solution (donkey anti-rabbit, IgG-conjugated Alexa FluorTM 488; 1:1,000; Molecular Probes, Monza, Italy). Slides were washed, cover-slipped with Vectashield mounting medium (Vector Laboratories, Peterborough, UK) and visualized under a Leica fluorescence microscope. 

### 4.7. Quantitative Image Analysis

The number of profiles positive for GFAP or Iba-1 were determined within a box measuring 10^4^ μm^2^ in the lateral, central and medial areas of the dorsal horn spinal cord sections, in the ipsilateral or contralateral sides. Eight L5 spinal sections were assessed from each of three animals per group, and a mean value obtained by combining values from lateral, central and medial areas of dorsal horn. To avoid overcounting cells, only cells counterstained with bisbenzimide were considered as positive profiles. 

### 4.8. Treatments

A total of 100 adult male mice were used. Behavioural, biomolecular and immunohistochemical studies have been performed on the same animals used first for behavioural observations and then sacrificed and dissected for biomolecular and immunohistochemical analysis. Mice were chronically (3 and 7 days) treated and grouped (n = 6) as follows: sham mice treated with vehicle (0.5% DMSO in 0.9% NaCl, i.p.); sham mice treated with 5′Cl5′d-(±)-ENBA (0.1, 0.5, 1 mg/Kg/day, i.p.); SNI mice treated with vehicle; SNI mice treated with 5′Cl5′d-(±)-ENBA (0.1, 0.5, 1 mg/Kg/day, i.p.) alone or 5′Cl5′d-(±)-ENBA (0.5, mg/Kg/day, i.p.) in combination with DPCPX (3 mg/Kg); sham mice treated with DPCPX; SNI mice treated with DPCPX. Another group of SNI mice (n = 6) received a single 5′Cl5′d-(±)-ENBA administration 7 days after surgery. DPCPX was administered 5 min before 5′Cl5′d-(±)-ENBA. Pre-treatment times and doses of antagonists were chosen based on our previous work in rodents [15,18].

### 4.9. Drugs

5′Cl5′d-(±)-ENBA was synthesized by Mario Grifantini’s team at the University of Camerino, Italy. 8-Cyclopentyl-1,3-dipropylxanthine (DPCPX) was purchased from Tocris Cookson Ltd. (Bristol, UK). Drugs were dissolved in 0.5% DMSO in saline for intraperitoneal administrations.

### 4.10. Statistical Analysis

Behavioural data are represented as means ± S.E.M. Repeated measure two-way ANOVA, followed by Student Neuman–Keuls *post hoc* test were used to determine the statistical significance among groups. Immunhistochemical data are represented as means ± S.E.M. ANOVA, followed by Tukey’s *post hoc* test. *p* < 0.05 was set as the level of statistical significance. Molecular data are shown as means ± S.E.M. and analysed by ANOVA, followed by Student–Neuman–Keuls *post hoc* test, *p* < 0.05 was considered statistically significant.

## 5. Conclusions

In conclusion, this study provides evidence that A_1_AR agonist 5′Cl5′d-(±)-ENBA, at a dose which does not affect the blood pressure, is able to prevent mechanical allodynia and thermal hyperalgesia, without exerting any significant effect on motor coordination, in the SNI model of neuropathic pain in mice. Moreover, *ex vivo* evaluations revealed that 5′Cl5′d-(±)-ENBA reduced the number of activated glial and microglial cells in the ipsilateral dorsal horn of SNI mice. These data indicate that A_1_ receptor may represent another target for glial-mediated purinergic control involved in the spinal plasticity occurring in the establishment and maintenance of neuropathic pain, although its precise role on astrocytes, as well as perhaps on microglia, needs further investigation. The encouraging results obtained with 5′Cl5′d-(±)-ENBA suggest that this compound may be a valid candidate for the preclinical development of a new drug for the treatment of neuropathic pain.

## Supplementary Materials

Supplementary Materials can be accessed at: http://www.mdpi.com/1420-3049/17/12/13712/s1.
