# Supplementary material for: 5'-Chloro-5'-deoxy-(±)-ENBA, a Potent and Selective Adenosine A1 Receptor Agonist, Alleviates Neuropathic Pain in Mice Through Functional Glial and Microglial Changes without Affecting Motor or Cardiovascular Functions
Source: Molecules. 2012 Nov 22;17(12):13712–26. doi: 10.3390/molecules171213712 (PMC6268894; doi:10.3390/molecules171213712)
Supplement: Supplementary file 1 [file molecules-17-13712-s001.pdf]

Supplementanr Material

Figure 1. Sequence alignment of the human (ADORA1-1) and murine (Adora1-1) A_1ARs. The sequence alignment was performed with the program MultiAlign.

```
           1                                                                                                                                130
ADORA1-1   MPPSISAFQAAYIGIEVLIALVSVPGNVLVIWAVKVNQALRDATFCFIVSLAVADVAVGALVIPLAILINIGPQTYFHTCLMVACPVLILTQSSILALLAIAVDRYLRVKIPLRYKMVVTPRRAAVAIAG
Adora1-1   MPPYISAFQAAYIGIEVLIALVSVPGNVLVIWAVKVNQALRDATFCFIVSLAVADVAVGALVIPLAILINIGPQTYFHTCLMVACPVLILTQSSILALLAIAVDRYLRVKIPLRYKTVVTQRRAAVAIAG
Consensus  MPPsISAFQAAYIGIEVLIALVSVPGNVLVIWAVKVNQALRDATFCFIVSLAVADVAVGALVIPLAILINIGPQTYFHTCLMVACPVLILTQSSILALLAIAVDRYLRVKIPLRYKmVVTqRRAAVAIAG

           131                                                                                                                              260
ADORA1-1   CWILSFVVGLTPMFGWNNLSAVERAWAANGSMGEPVIKCEFEKVISMEYMVYFNFFVWVLPPLLLMVLIYLEVFYLIRKQLNKKVSASSGDPQKYYGKELKIAKSLALILFLFALSWLPLHILNCITLFC
Adora1-1   CWILSLVVGLTPMFGWNNLSEVEQAWIANGSVGEPVIKCEFEKVISMEYMVYFNFFVWVLPPLLLMVLIYLEVFYLIRKQLNKKVSASSGDPQKYYGKELKIAKSLALILFLFALSWLPLHILNCITLFC
Consensus  CWILSlVVGLTPMFGWNNLSaVErAWaANGSmGEPVIKCEFEKVISMEYMVYFNFFVWVLPPLLLMVLIYLEVFYLIRKQLNKKVSASSGDPQKYYGKELKIAKSLALILFLFALSWLPLHILNCITLFC

           261                                                              326
ADORA1-1   PSCHKPSILTYIAIFLTHGNSAMNPIVYAFRIQKFRVTFLKIWNDHFRCQPAPPIDEDLPEERPDD
Adora1-1   PTCQKPSILIYIAIFLTHGNSAMNPIVYAFRIHKFRVTFLKIWNDHFRCQPKPPIEEDIPEEKADD
Consensus  PsCqKPSILiYIAIFLTHGNSAMNPIVYAFRIqKFRVTFLKIWNDHFRCQPaPPI#EDiPEEraDD
```
